# Lipid Dysregulation and Impaired Resolution of Inflammation in Genetic Forms of Parkinson’s Disease: Potential Links and Knowledge Gaps

**DOI:** 10.3390/biomedicines14102305

**Published:** 2026-10-10

**Authors:** Tatiana Gerasimova, Ekaterina Novosadova, Ekaterina Stepanenko, Artem Eremeev, Valentina Nenasheva

**Affiliations:** 1 Laboratory of Translational Biomedicine, Lopukhin Federal Research and Clinical Center of Physical–Chemical Medicine of Federal Medical Biological Agency, 1A Malaya Pirogovskaya Str., 119435 Moscow, Russia; novek-img@mail.ru (E.N.); katishsha@mail.ru (E.S.); val-nenasheva@mail.ru (V.N.); 2 Center for Genetic Reprogramming and Gene Therapy, Lopukhin Federal Research and Clinical Center of Physical–Chemical Medicine of Federal Medical Biological Agency, 1A Malaya Pirogovskaya Str., 119435 Moscow, Russia; art-eremeev@yandex.ru

**Keywords:** Parkinson’s disease, PD-associated genes, lipid metabolism, specialized pro-resolving mediators

## Abstract

Dysregulation of lipid metabolism plays an important role in the development of Parkinson’s disease (PD), particularly in PD monogenic forms. At the same time, accumulating evidence points to impaired resolution of neuroinflammation mediated by specialized pro-resolving mediators (SPMs), although mutation-specific data remain limited. Mutations in PD-associated genes, such as *GBA1*, *LRRK2*, *SNCA*, *PLA2G6* and others, are linked to lipidomic changes, including altered sphingolipid, phospholipid, and polyunsaturated fatty acid profiles in plasma membranes, organelle membranes, and extracellular vesicles. These disturbances influence membrane composition, fluidity, and dynamics, and may also affect the biosynthesis and activity of SPMs. Importantly, the nature of lipid metabolism dysregulation appears to be mutation-specific and may differentially impact SPM biosynthetic pathways and the availability of SPM receptors, and downregulate SPM signaling, leading to heterogeneous inflammatory phenotypes across genetic forms of PD. Here, we attempt to trace potential links between lipid metabolic dysfunction and disruption of SPM functioning in genetic PD, with a focus on mutation-specific changes. This integrated view may provide new insights into PD mechanisms and guide the development of mutation-specific therapeutic approaches aimed at restoring pro-resolving lipid signaling in genetically defined forms of the disease.

## 1. Introduction

Parkinson’s disease (PD) can be seen as a lipid-induced proteinopathy, where changes in lipid composition and metabolism cause α-synuclein (α-Syn) misfolding and neuronal loss [1]. The endosome/lysosome system and synaptic signaling dysregulated in PD critically depend on lipid dynamics [2]. Correlative light and electron microscopy indicates that Lewy bodies are rich not only in α-Syn but also in lipids, membrane fragments and membranous organelles, including vesicles [3,4]. Polyunsaturated fatty acids (PUFAs) such as eicosapentaenoic (EPA) and docosahexaenoic (DHA) are essential dietary fatty acids that mammals cannot synthesize de novo. Experimental models show that DHA, a major neuronal membrane fatty acid, readily promotes α-Syn aggregation, and the morphology of aggregates depends on the protein:DHA ratio [5]. α-Syn also feeds back into lipid metabolism: its expression alters acyl-CoA synthetase activity and localization in the endoplasmic reticulum, changing arachidonate turnover in brain phospholipids. α-Syn–PUFA interactions regulate neuronal PUFA levels [6]. It was shown that in α-Syn knockout mice, not only were DHA incorporation and turnover increased, but changes in lipid metabolism were more complex: cholesteryl esters and cholesterol were higher, and cardiolipin (CL) and phosphatidylglycerol were reduced [1,7]. More than 20 lipid metabolism-related PD characteristic genes were identified [8].

Experimental studies support the involvement of PD-linked genes in lipid metabolism. Evidence exists showing that such genes as *GBA1*, *SMPD1*, *LRRK2* (*PARK8*), *SYNJ1* (*PARK20*), *PLA2G6* (*iPLA2β*, *PARK14*), *PARK2*, etc. regulate sphingolipid degradation, vesicular lipid trafficking, phospholipid remodeling, fatty acid uptake, and mitochondrial lipid homeostasis [1,2,9,10,11]. These lipid pathways define the availability, compartmentalization and turnover of PUFAs in neuronal and glial membranes, including the omega-3 (ω-3) substrates from which specialized pro-resolving mediators (SPMs) are generated. In parallel, studies in experimental PD models and in patients have reported reduced SPM levels, together with alterations in the expression of SPM receptors, compared with healthy controls [12]. These two lines of evidence together suggest that genetic disruption of lipid handling in PD appears to converge on impaired resolution signaling. Moreover, an association between higher ω-3 PUFA intake and a lower risk of PD has been reported in the literature, including a recent systematic review on effects of ω-3 PUFAs on the brain lipid profile and neuroinflammation in various rodent experimental models of PD [13,14]. It is well established that anti-inflammatory eicosanoids are among the end products of ω-3 fatty acid metabolism. They inhibit cytokine production and directly counteract the effects of pro-inflammatory prostaglandins and leukotrienes [15], highlighting the close relationship between lipid homeostasis and the resolution of inflammation, a process mediated in part by SPMs. A logical next step is to move beyond the observation of reduced SPM levels in PD and determine how dysfunction of individual PD-associated genes affects SPM biosynthesis, distribution, and receptor-mediated signaling. Although gene-specific measurements of SPMs are still lacking, therapeutic strategies for PD should be mutation-specific [16].

Taken together, these observations suggest that resolution failure in genetic forms of PD does not occur in isolation, but is dependent on a genetically altered lipid metabolism.

Previous reviews have addressed SPMs and their therapeutic potential in neurodegenerative disorders [17], while other studies have focused on lipid dysregulation in PD and its relationship with α-synuclein [18]. The interaction between α-synuclein (α-Syn) and different classes of bioactive lipids, including SPMs, has also been reviewed [19]. A recent review by Li et al. (2026) discussed pro-resolving lipid mediators in different diseases, including PD, and suggested that promoting the resolution of inflammation, rather than simply suppressing inflammation, may represent a promising therapeutic approach [20]. However, the potential relationship between individual PD-associated genetic alterations, their specific effects on lipid metabolism, and subsequent changes in the SPM system has not been systematically addressed. This represents a gap in current knowledge and provides the basis for exploring these potential links in the present review.

The literature search was carried out in PubMed in peer-reviewed sources, tracing relevant studies up to and including 2026. No lower publication date limit was applied, as some findings reported in earlier studies remain important for the topics discussed in this review. The primary search was focused on PD-linked genes and their involvement in lipid metabolism using keywords such as names of PD-linked genes, “lipids”, “PUFA”, “lipid metabolism”, “lipid transport”, and “membrane lipid composition”. Further, a literature search was conducted for each of the genes, focusing on their roles in PD. Additional searches included terms related to SPM, including “resolvins”, “protectins”, “maresins”, “SPM biosynthesis”, and “resolution of inflammation”, alone or in combination with “Parkinson’s disease” and individual PD-associated genes.

## 2. Lipid Alterations in Genetic Forms of PD

Accumulating evidence indicates that multiple levels of lipid homeostasis are disrupted in PD, such as membrane phospholipid and sphingolipid composition and the activity of individual enzymes. Several PD-associated genes directly affect lipid metabolism, and others act on common lipid-modifying pathways that influence dopaminergic neurons. To the best of our knowledge, there are no data on the direct impact of mutations in PD-associated genes on the SPM system; therefore, we have made an attempt to draw possible conceptual links to highlight potential directions for future research (Table 1 and Table 2).

Changes in lipid metabolism in PD are observed in plasma membrane, mitochondrial and lysosomal membranes, components of endosomal traffic and the extracellular vesicle system. According to available data, these changes are associated with mutations in certain PD-related genes.

Total cholesterol level and membrane fluidity are altered by Parkin deficiency and may be a causal factor of the disease [50]. A recent work has established membrane lipid dysregulation as a core pathogenic mechanism in *LRRK2*-associated PD, where GTPase-domain mutations such as I1371V selectively deplete cholesterol from the neuronal plasma membrane, increase membrane fluidity, and disrupt lipid microdomains [16]. Loss-of-function *GBA1* mutations such as L444P lead to a distinctly altered membrane lipid profile in PD patient fibroblasts, particularly in sphingolipid and phospholipid classes, providing a lipidomic fingerprint that likely promotes α-Syn aggregation [3]. Mechanistic research shows that Parkin mono-ubiquitinates the class B scavenger receptor CD36/FAT, thereby stabilizing it at the plasma membrane and promoting uptake of long-chain FA and lipoprotein-derived lipids [10]. Lipid disruptions at the plasma membrane could interfere with lipid–protein interactions and affect intracellular signaling responses, including inflammatory pathways.

Genetic and experimental data support the involvement of autophagy–lysosomal pathway dysregulation in PD [1]. *GBA1* and *ATP13A2* mutations are involved in disease-associated processes. In a recent study, the consequences of reduced GCase activity were investigated, focusing primarily on the lysosomal compartment and showing alteration of lysosomal membrane stability and intralysosomal accumulation of sphingolipids and cholesterol [50]. Loss of the enzyme function in patient dopaminergic neurons and in GBA1 models led to accumulation of glucosylceramide, the lipid able to strongly interact with α-Syn and accelerate its aggregation [22]. In a PD brain, the activity of other sphingolipid hydrolases is also reduced; thus, their sphingolipid substrates accumulate, while gangliosides decline [51]. PD-associated ATP13A2 is a lysosomal P5B-ATPase that lowers the levels of the anionic phospholipid bis(monoacylglycero)phosphate. Overexpression of the gene in the SH-SY5Y cell line decreases the total content of triglycerides (TGs), cholesterol and lipid droplets. As TGs are necessary for the synthesis of new membranes, this observation suggests that increased function of ATP13A2 switches the endolysosomal system toward vesicle secretion [52]. Together these findings indicate broad changes in lysosomal lipid metabolism associated with mutations in specific PD-associated genes.

Lipidomic analysis of mitochondria from aged *Park2*-null mice reveals decreased mitochondrial phospholipids levels and CL degree of unsaturation [25]. The mitochondrial lipid dysregulation in PD involves PLA2G6, PLD1/PLD2 isoforms and Parkin/PINK1. PLA2G6 is a group VI calcium-independent phospholipase A2β that hydrolyses glycerophospholipids to liberate arachidonic acid and other fatty acids, and mutations in this gene are implicated in PD [1]. Parkin is also involved in regulation of FA uptake and mitochondrial lipid composition. PINK1 acts in concert with Parkin to mediate mitochondrial quality control and mitochondria-derived vesicle formation [53]. In mitochondria, Parkin coordinates lipid remodeling during mitophagy by recruiting PLD2 and is required for the activation of PA phosphatase Lipin-1 on the outer mitochondrial membrane [42].

The endosomal trafficking is regulated by LRRK2, Rab GTPases and synaptojanin 1. Rab GTPases phosphorylated by LRRK2 regulate lipid droplet biogenesis, fusion and mobilization, and they organize lipid droplet contact sites with mitochondria, endosomes, peroxisomes and the plasma membrane [2]. Along the endocytic pathway, LRRK2 also regulates clathrin adaptor protein complex 3 (AP-3), a key mediator of intracellular recycling of membrane proteins that is extensively used by neurons. Auxilin and SYNJ1 act at sequential stages of synaptic vesicle endocytosis. During vesicle formation, clathrin and its adaptor proteins remodel the presynaptic plasma membrane. Following vesicle budding, auxilin functions as an Hsc70 co-chaperone to promote clathrin coat disassembly. SYNJ1 cooperates with auxilin during this uncoating step and supports efficient synaptic vesicle recycling. Loss-of-function mutations in SYNJ1, as a downstream substrate of LRRK2, disrupt the phosphoinositide homeostasis and lead to defects in vesicle recycling and early endosomal compartments [54]. In patients, mutations in *DNAJC6* lead to early-onset autosomal recessive parkinsonism with clinical symptoms highly similar to PD [34], and mutations in the Rab39b gene cause atypical PD [55]. Significant accumulation of both LAMP1 and LAMP2 at the cell surface was observed in HEK293 cells treated with *LRRK2* siRNA or *AP3B1* siRNA, compared with the control [22], suggesting that *LRRK2* mutations may influence intracellular trafficking and lysosomal exocytosis. Interestingly, SYNJ1 is a PA-binding phosphoinositide phosphatase that preferentially associates with PUFA-containing PAs which enhance the activity of its Sac1 (4-phosphatase) domain [56]. This mechanism demonstrates that PUFA-containing PAs act as functional regulators of SYNJ1, directly linking membrane lipid composition to vesicle formation.

Since endosomal and endolysosomal trafficking is tightly coupled to the biogenesis and release of extracellular vesicles, alterations in this pathway are expected to impact both quantity and molecular composition of exosomes, which are important players in astrocyte-to-neuron communications. Impaired exosome release and cargo are known to be involved in the pathogenesis of PD. The lipid architecture influences their bioactivity and supports structural rigidity and stability [57]. In PD patients, *PLA2G6* mutations have been linked to altered lipid profiles of CSF-derived exosomes [11], consistent with the role of phospholipase A2. Mutations in other PD-associated genes, such as *GBA1* [58] and *LRRK2* [47], have also been reported to modify the concentration, size and molecular composition of circulating exosomes and small extracellular vesicles. In dopaminergic neurons, Parkin regulates lipid homeostasis through a Parkin/SREBP2/lipoprotein lipase axis, which controls lipid droplet formation under PD-linked oxidative stress. Activation of this pathway supports an adaptive lipid droplet response [26]. Interestingly, EVs can carry PUFAs [59] that are precursors for SPM biosynthesis, providing a potential link between genetic perturbations of lipid handling, EV cargo and SPM-related signaling.

Taken together, in genetic forms of PD, mutations in PD-associated genes give rise to diverse disturbances of lipid homeostasis, which converge on the SPM pathway and are likely to impair its function at several levels. However, data directly linking these two aspects are currently lacking in the literature. We have attempted to trace possible connections (Figure 1).

## 3. SPM System Dysfunction in Genetic Forms of PD

It should be emphasized that mutations in PD-related genes support neuroinflammation. Gain-of-function mutations in *LRRK2* and *SNCA* and loss-of-function mutations in *GBA1* promote pro-inflammatory signaling. Mutations in *PINK1* and *PARK2* also trigger inflammation via activation of NLRP3 inflammasome and the release of mitochondrial DNA [60]. Direct evidence that PD-linked mutations specifically alter pro-resolving lipid mediator biosynthesis or signaling is currently lacking. Nevertheless, it is clear that the amount and availability of substrate, the activity of enzymes involved in SPM biosynthesis, and the presence of the receptors to these ligands on the membranes of target cells may directly influence the functioning of the SPM system.

### 3.1. Disrupted Substrate Availability for SPM Synthesis

Once incorporated into membrane phospholipids and lipid droplets, PUFAs provide the substrate pool for SPM biosynthesis, predominantly in astrocytes and microglia [12]. Astrocytes and endothelial cells cooperate to supply ω-3 fatty acid precursors and DHA to neurons [61]. Formation of PUFA-derived lipid mediators is initiated by membrane-associated phospholipase A2, which releases PUFAs from phospholipids. Therefore, membrane lipid composition is an important determinant of substrate availability for lipid mediator biosynthesis and has been shown to be altered in PD. Lipid rafts from human PD cortices contained reduced levels of n-3 and n-6 long-chain PUFAs, DHA, AA, and DPA. At the same time, saturated fatty acids were significantly increased compared with age-matched controls [62]. Moreover, increased lipid peroxidation was detected in the substantia nigra of patients with advanced-stage PD compared with healthy controls [63]. Loss of synaptojanin 1 function causes PI(4,5)P_2_ accumulation, impairing phosphoinositide turnover [31], and upregulated α-Syn modulates acyl-CoA synthetase activity and affects turnover of arachidonate [1,7]. Metabolomic screening in *PARK14* models showed that *PLA2G6* mutations were associated with reduced phospholipase activity, increased phospholipid levels, and decreased DHA levels [24]. However, the effects of *PLA2G6* mutations appear to depend on the specific mutation. While mutations associated with INAD can result in a marked loss of PLA2G6 catalytic activity, mutations associated with dystonia-parkinsonism may affect other aspects of PLA2G6 function, including substrate preferences or regulatory mechanisms [64]. Thus, different alterations in PLA2G6 function may be associated with changes in phospholipid and fatty acid metabolism and membrane lipid composition. Together, these reports suggest that dysfunction of PD-associated genes may alter membrane lipid and fatty acid metabolism, potentially affecting the availability of DHA and EPA for SPM production.

Unique to mitochondria, CL constitutes up to 20% of mitochondrial inner membrane and is essential for mitochondrial membrane functioning. It is established that DHA and EPA provide fluidity and other essential properties to the lipid bilayer, including signaling properties of cellular receptors [65]. The molecular identity and stereospecificity of PUFAs in neuro-CLs was investigated demonstrating that fatty acids were mostly represented by AA, while DHA and EPA were detected in low levels. PINK1 and Parkin, two major proteins associated with familial PD, are involved in mitochondrial quality control and are also linked to lipid homeostasis. A direct link between PD-associated genes, fatty acid metabolism, and mitochondrial membrane lipid composition has been demonstrated. In aged Parkin-null mice, brain mitochondria showed decreased levels of phosphatidylglycerol and phosphatidylinositol and increased levels of some hydroxylated ceramides. Alterations in CL unsaturation were also observed, although these changes were mainly associated with aging rather than Parkin deletion [24 Gaudioso]. In PINK1-deficient models, including patient-derived fibroblasts and iPSC-derived dopaminergic neurons, inhibition of fatty acid synthase (FASN) reduced palmitate levels and increased CL, a lipid specific to the inner mitochondrial membrane. Importantly, increasing CL levels rescued PINK1-associated complex I defects, while direct CL supplementation restored inefficient electron transfer between complex I and ubiquinone [27]. These findings indicate a close relationship between PARK2 and PINK1 deficiency, fatty acid metabolism, and mitochondrial membrane lipids. Although a direct relationship between PINK1/Parkin mutations and SPM biosynthesis has not been demonstrated, there is evidence that SPMs can modulate mitochondrial pathways involving these proteins. In a non-PD model of ischemia/reperfusion injury, RvD1 attenuated PINK1 accumulation and restored reduced Parkin protein levels. RvD1 also decreased mitochondrial lipid peroxidation and restored the activities of mitochondrial complexes I and III [66]. Thus, mitochondria do not appear to represent a site of SPM biosynthesis, but mitochondrial lipid homeostasis and PINK1/Parkin-dependent quality-control pathways may represent points of interaction between PD-associated mechanisms and SPM signaling.

Typically, modifications in lipid composition lead to downstream alterations in intracellular signaling pathways [67]. Abnormalities of lipid rafts in the frontal cortex occur at early stages of PD, correlating with abnormal lipid raft signaling [62]. Such alterations could potentially affect SPM-receptor signaling.

### 3.2. Altered Activity of Enzymes Involved in SPM Production

PLA2G6 releases DHA from membrane phospholipids and may supply DHA for the biosynthesis of DHA-derived SPMs [68]; then, two lipoxygenases, 15-LOX and 5-LOX, participate sequentially in these biosynthetic pathways. The activity of another implicated enzyme, COX-2, commonly causes an excessive production of pro-inflammatory prostaglandins (PGE2) [69], but at the same time, PGE2 is necessary for the switch to SPM synthesis and timely resolution of inflammation [70]. PLA2G6-mediated release of DHA may provide substrate for 15-LOX- and 5-LOX-dependent biosynthetic pathways, leading to RvD1 formation [71]. Parkin deficiency decreased LPS-induced COX-2 expression in the RAW 264.7 murine macrophage-like cell line, and human fibroblast-like synoviocytes derived from patients with rheumatoid arthritis [72]. In primary dermal fibroblasts from patients with two common mutations in LRRK2 (G2019S and R1441G), COX-2 RNA levels were very high compared to age-matched healthy individuals [39]. DA neurons differentiated from patients with G2019S LRRK2 mutation showed increased COX-2 expression following MPP^+^ exposure [73]. *LRRK2* silencing significantly reduced basal COX-2 levels and COX-2 induction after a pro-inflammatory stimulus [39], which correlates with the fact that in PD, *LRRK2* expression is upregulated.

In a recent study, the reverse influence was demonstrated: LOX-15 increased LRRK2 phosphorylation and kinase activity, acting as an upstream regulator of the *LRRK2* pathogenic hyperactivation [74]. Similarly, selective COX-2 inhibitor parecoxib increased PINK1/Parkin levels, activating mitophagy [75]. To our knowledge, there is currently no direct evidence that mutations in other PD-associated genes (such as *SNCA*, *GBA1*) specifically alter the expression or catalytic activity of the enzymes involved in SPM synthesis, although changes in SPM levels have been reported in PD models and patients [12,76].

### 3.3. Dysfunction of SPM Receptors

SPMs act via G protein-coupled receptors (GPCRs) on the plasma membrane, including ALX/FPR2, CHEMR23, GPR18 and GPR37, which are broadly expressed on brain cells of different types. Through these receptors, lipoxins, resolvins, protectins and maresins activate intracellular signaling pathways and act as pro-resolving mediators [77].

It has been shown that ALX/FPR2 is stored in secretory granules in cytoplasm of polymorphonuclear neutrophils, but is rapidly mobilized to the cell surface when the cells are exposed to pro-inflammatory stimuli [78]. The process of transporting receptors to the surface of the plasma membrane is clearly influenced by its lipid composition. Disrupted lipid raft integrity—for example, by cholesterol depletion—alters raft-dependent internalization mechanisms and the ability of the receptors to respond to stimuli [79]. For the lipoxin receptor ALX/FPR2, internalization and downstream signaling have been shown to depend on cholesterol-rich lipid rafts: disrupting these microdomains with filipin completely prevents receptor uptake [45]. This suggests that raft lipids, such as cholesterol and sphingolipids, are critical for endocytosis of at least some SPM-responsive GPCRs. Conversely, recent work investigated the effect of SPMs themselves on lipid raft integrity, and showed the ability of RvD1 and RvD2 to alter the membrane polarity related to cholesterol composition, as assessed by fluorescence spectroscopy [80].

Importantly, although several PD-associated genes have been directly linked to alterations in lipid metabolism, experimental evidence demonstrating that these changes subsequently affect SPM biosynthesis or signaling remains very limited. Thus, many of the proposed links between PD-associated gene dysfunction and the SPM system should currently be considered hypothetical and require direct experimental validation.

## 4. Conclusions

In genetic forms of PD, mutations in several key genes disturb lipids through distinct mechanisms that are likely to compromise SPM signaling at different levels. Among the analyzed PD-associated genes, however, several patterns can be identified. A first group of mutations, in *PLA2G6*, *SNCA*, *PARK2*, and *ACSL4* genes, predominantly reduce availability or enhance oxidation of DHA- and EPA-containing substrates required for SPM biosynthesis at mitochondrial and plasma membranes. Here, supplementation with SPMs and their precursors may be beneficial. In the case of *PARK2* and *PLD2* mutations, the changes affect the activity of lipid-metabolizing enzymes and associated pathways, which can shift AA, EPA and DHA cascades toward pro-inflammatory eicosanoids at the expense of pro-resolving mediators. A rational strategy might include not only SPM and precursor supplementation but also approaches that rebalance these enzymatic pathways, for example, taking advantage of aspirin-induced SPM production. A second group, including mutations in *GBA1*, *SMPD1*, and *ATP13A2*, is primarily linked to endolysosomal and vesicular compartments in which SPMs and their receptors operate. In this context, future strategies might include the use of engineered extracellular vesicles as carriers of SPMs or their precursors to defined cellular and subcellular targets to compensate impaired endogenous vesicular trafficking. By contrast, in the case of mutations in *LRRK2*, *SYNJ1*, and *DNAJC6*, which markedly affect lipid microdomains, endocytosis and Rab-dependent trafficking, the critical point is the availability and functioning of SPM receptors, including their internalization, recycling and location in membrane microdomains. Here, potentially relevant strategies would be the usage of agonists of SPM receptors and agents that stabilize receptors at the cell surface and support their recycling.

Taken together, different PD mutations produce not a uniform “resolution failure”, but rather distinct, specific disturbances of the SPM system. A logical next step is to obtain profiles of SPMs, their precursors, receptor expression and trafficking in patient-derived cells carrying mutations in different genes. Such studies will help to clarify how individual PD-associated mutations affect resolution of neuroinflammation through lipid metabolism alterations in genetic forms of PD, and to identify for each mutation which SPM or combination of SPMs and which level of the SPM pathway (SPMs themselves, their PUFA precursors, receptors or biosynthetic enzymes) are the most appropriate therapeutic target.

## Figures and Tables

**Figure 1:**
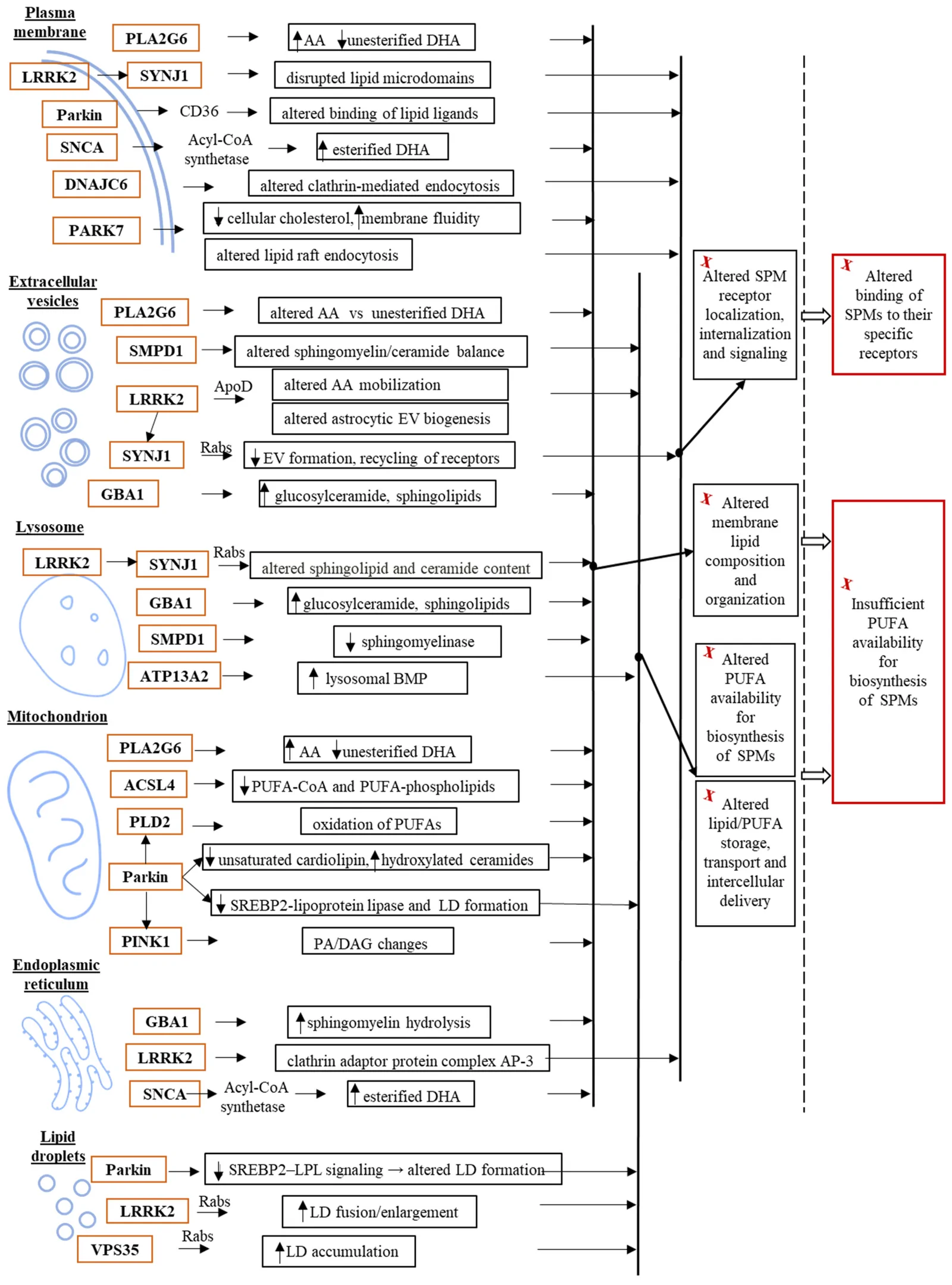
The influence of mutations in PD-associated genes on lipid metabolism with conceptual links to SPM system function. In the left part of the figure, published evidence on the effects of mutations in specific Parkinson’s disease-associated genes on lipid metabolism within distinct intracellular compartments is summarized. In the right part, we outline possible correlations between these alterations and impaired SPM-mediated resolution of inflammation. ↑, increase; ↓, decrease; red X, disruption of the indicated process.

**Table 1:** PD-associated genes with primary effects on lipid metabolism and potential impact on SPM.

PD-Associated Gene (Protein)	Gene-Associated Lipid Alterations	Experimental Model, Tissue or Cell Type, Mutation	Link to SPMs (Conceptual)
*GBA1* (glucosylceramidase beta 1)	↓ GCase activity; ↑ neuronal glucosylceramide and sphingolipids [21]	Primary mouse cortical neurons; GCase knockdown [21]	Altered membrane composition may affect DHA/EPA-containing pools for SPM biosynthesis and exosomal transport
*LRRK2* (leucine-rich repeat kinase 2)	G2019S: altered Rab phosphorylation and endolysosomal/autophagic trafficking [22] Synaptojanin 1 phosphorylation and disrupted synaptic vesicle trafficking [23] Altered sterols, sphingolipids, phospholipids, fatty acyls and glycerolipids [2]	*C. elegans* lrk-1(km17) and human LRRK2 WT/G2019S transgenic lines, motor neuron axons [22] G2019S transgenic mouse midbrain neurons [23] CSF and serum from PD patients and G2019S carriers [2]	Endolysosomal and LD alterations may redistribute DHA/EPA-containing phospholipids and affect SPM precursor/receptor localization
*PLA2G6* (phospholipase A2 group VI)	Altered exosome structure and exosomal lipidome [11] ↓ phospholipase activity; ↑ phospholipids ↓ DHA [24]	CSF exosomes from PD patients carrying PLA2G6 mutations (c.1966C>G, Leu656Val; c.2077C>G, Leu693Val; c.1791delC, His597fx69) and SMPD1 mutations [11] Zebrafish expressing PLA2G6 D331Y/T572I PLA2G6 D331Y knock-in mice [24]	Imbalanced release of arachidonic acid versus ω-3 PUFAs (DHA/EPA) from neuronal membranes may limit substrate availability for SPM biosynthesis
*PARK2* (Parkin)	↑ less-unsaturated CL; ↓ phosphatidylglycerol and PI ↑ some hydroxylated ceramides [25] SREBP2–LPL-dependent regulation of neuronal lipid homeostasis and LDs [26] Stabilization of lipid-binding CD36 [10]	Parkin-null mouse mitochondrial membranes (24 months) [25] Parkin T240R SH-SY5Y cells; Parkin exon-7 deletion mice, whole brain and primary cortical neurons [26] Parkin−/− mice, high-fat/high-cholesterol diet, whole brain [10]	Altered LD/FA trafficking may affect DHA/EPA mobilization for SPM biosynthesis; CD36 destabilization may impair long-chain FA, oxidized phospholipid and PUFA handling
*PINK1* (PTEN-induced kinase 1)	↓ FASN activity and palmitate; ↑ CL [27] ↑ mitochondrial ceramide; ↓ FA β-oxidation [28]	MEF mitochondrial fractions [27] PINK1-mutant flies, Pink1−/− MEFs and fibroblasts from patients with homozygous *PINK1* mutations [28]	Altered mitochondrial lipid remodeling and turnover may influence local PUFA availability and oxidation
*SMPD1* (sphingomyelin phosphodiesterase 1)	↓ acid sphingomyelinase activity; altered EV formation/secretion and sphingomyelin/ceramide balance [11] Mutant enzyme retained in ER rather than lysosomes [29]	CSF exosomes from PD patients with SMPD1 G508R [11] *SMPD1* KO/knockdown (p.L302P, p.fsP330) in HeLa and BE(2)-M17 dopaminergic cells [29]	Disturbed sphingomyelin/ceramide homeostasis and lysosomal function may remodel PUFA-enriched membranes involved in SPM generation/action
*SNCA* (alpha-synuclein, α-Syn)	*SNCA* deletion increases DHA incorporation and turnover in brain phospholipids [7] α-Syn-duplication neurons showed an increase in the concentration of PS as well as all species of phospholipids bound to PUFAs [30]	Brains of *Snca*−/− mice [7] α-Syn-duplication human iPSC-derived neurons compared with control and α-Syn-KO neurons [30]	α-Syn excess alters PUFA membrane partitioning, potentially affecting substrate availability for SPM biosynthesis
*SYNJ1* (synaptojanin 1)	*SYNJ1* deficiency:PI(4,5)P_2_ accumulation in midbrain neurons, indicating impaired phosphoinositide turnover [23] SYNJ1 is considered a functional LRRK2 interactor [31]	*Synj1*+/− mice and primary midbrain neurons [23] Midbrain neurons from P0-P1 mice pups [31]	Altered PI(4,5)P_2_ homeostasis may affect phospholipase-dependent PUFA mobilization and substrate availability for SPM biosynthesis
*VPS13C* (VPS13C lipid transport protein)	*VPS13C* depletion: lysosomal accumulation and altered lipid profile, including PUFA-containing lipids [32]	WT and *VPS13C*-KO HeLa cells [32]	Altered EPA/DHA-containing lipid pools may affect substrate availability for SPM biosynthesis
*VPS35* (VPS35 retromer complex component)	*VPS35* knockdown: ↑ LD accumulation; *VPS35* overexpression: ↓ LD formation and inflammatory response [33]	Primary mouse microglia [33]	Altered lipid storage and mobilization may affect PUFA availability for SPM biosynthesis
*PARK7* (parkinsonism-associated deglycase DJ-1)	DJ-1 deficiency: ↓ cellular cholesterol, ↑ membrane fluidity and disrupted lipid raft-dependent endocytosis [34] ↓ *PTGDS* expression and PGD_2_ production [35]	*DJ-1* WT and KO MEFs [34] *DJ-1*-KO rat brain and astrocytes [35]	Altered membrane lipid organization and lipid mediator production may affect pro-resolving signaling
*ATP13A2* (lysosomal P5B-ATPase)	ATP13A2 loss: ↑ lysosomal BMP and neuronal LD accumulation [36]	Atp13a2-null mice, cerebral cortex [36]	BMP is preferentially enriched in DHA, altered BMP homeostasis may affect endolysosomal distribution of DHA

↑, increase; ↓, decrease.

**Table 2:** Lipid-modifying enzymes and pathways that intersect with PD gene-associated lipid changes and may influence SPM.

Enzyme/Pathway (Relation to PD Gene)	Enzyme-Associated Lipid Alterations	Experimental Model, Tissue or Cell Type, Mutation	Link to SPMs (Conceptual)
Acyl-CoA synthetase long-chain family member 4 (ACSL4) (associated with LRRK2-linked lipid oxidation; functionally linked to Parkin)	↑ ACSL4 and lipid peroxidation in SN dopaminergic neurons of homozygous LRRK2 N1437D mice [37] Parkin promotes ACSL4 ubiquitination/degradation and modulates PUFA-phospholipid metabolism [38]	Aged LRRK2 N1437D knock-in mice; SN TH^+^ neurons [37] H9c2, neonatal rat cardiomyocytes and HEK293T cells [38]	Altered ACSL4-dependent incorporation of PUFAs into phospholipids may affect PUFA availability for SPM biosynthesis
COX-2/LOX15 (PUFA-derived lipid mediator biosynthesis)	↑ COX-2 in LRRK2 G2019S/R1441G PD fibroblasts, reduced by *LRRK2* silencing [39] A53T α-Syn → ↑ ALOX15; ↑ ALOX15/PEBP1 complex formation and phospholipid peroxidation in chronic stress [40]	Dermal fibroblasts from PD patients carrying LRRK2 G2019S or R1441G mutations; lentiviral *LRRK2* silencing [39] SNCA A53T transgenic mice, midbrain; *Alox15*−/− mice with AAV-mediated A53T α-Syn expression [40]	Altered COX/LOX-dependent PUFA metabolism and phospholipid oxidation may influence PUFA substrate availability and pathways involved in SPM biosynthesis
Phospholipase D PLD2 (functionally interacting with α-synuclein and Parkin)	PLD2 lipase activity and PA production are associated with dopaminergic neurodegeneration [41] Parkin recruits PLD2 to damaged mitochondria, driving PA-to-DAG remodeling and accumulation of long-chain unsaturated DAG species during mitophagy [42] D-series resolvins activate PLD2, contributing to resolvin-induced pro-resolving action [43]	AAV-mediated WT or mutant PLD2 overexpression in rat SNc [41] HeLa cells expressing Parkin and SH-SY5Y cells with endogenous Parkin [42] Macrophages differentiated from PBMC [43]	PLD2 may provide a functional link between PD-associated lipid remodeling and D-series resolvin signaling
Auxilin (functionally linked to synaptojanin 1)	DNAJC6/Auxilin mutation reduces LC-PUFA-containing membrane glycerolipids/glycerophospholipids, particularly PI species; Synj1 partially restores these changes [44] Auxilin regulates clathrin-mediated endocytosis; FPR2/ALX undergoes clathrin-dependent internalization/recycling required for LXA_4_-induced pro-resolving actions [45]	*Drosophila* Auxilin R1119G knock-in (human R927G equivalent) ± neuronal Synj1 overexpression [44] HeLa cells [45]	Auxilin-dependent alterations in membrane lipids and endocytic trafficking may affect PUFA availability and FPR2/ALX-mediated pro-resolving signaling
Rab8a (LRRK2 phosphorylation substrate)	LRRK2 Y1699C phosphorylates Rab8a at T72, promoting lipid droplet fusion and enlargement [46]	3T3-L1 cells expressing LRRK2 Y1699C and Rab8a WT/T72 mutants [46]	LRRK2/Rab8a-dependent changes in lipid droplet dynamics may affect cellular PUFA trafficking and availability for SPM biosynthesis
ApoD/astrocyte-derived extracellular vesicles (EVs)	LRRK2 G2019S alters astrocytic EV biogenesis and EV-mediated support of dopaminergic neurons [47] Astroglial EVs transfer ApoD to neurons [48] Association of ApoD with increase in AA, EPA, and DHA [49]	LRRK2 G2019S patient-derived and gene-corrected iPSC astrocytes/DA neurons [47] 1321N1 astroglia/SH-SY5Y neurons [48] ApoD-overexpressing mouse model of LPS-induced sepsis [49]	PD-associated alterations in astrocytic EVs may affect ApoD-mediated AA availability for lipoxin biosynthesis

↑, increase.

## Data Availability

No new data were created or analyzed in this study. Data sharing is not applicable to this article.

## Abbreviations

The following abbreviations are used in this manuscript:

AA	arachidonic acid
ACSL4	acyl-CoA synthetase long-chain family member 4
AP-3	adaptor protein complex 3
CL	cardiolipin
COX-2	cyclooxygenase-2
DAG	diacylglycerol
DHA	docosahexaenoic acid
DNAJC6	DnaJ heat shock protein family (Hsp40) member C6
EPA	eicosapentaenoic acid
EV	extracellular vesicle
GBA1	glucosylceramidase beta 1
GCase	glucocerebrosidase
GPCR	G protein-coupled receptor
INAD	infantile neuroaxonal dystrophy
iNOS	inducible nitric oxide synthase
LOX	lipoxygenase
LRRK2	leucine-rich repeat kinase
PA	phosphatidic acid
PD	Parkinson’s disease
PGE2	prostaglandin E2
PINK1	PTEN-induced kinase 1
PLA2G6	phospholipase A2 group VI
PLD	phospholipase D
PUFAs	polyunsaturated fatty acids
SMPD1	sphingomyelin phosphodiesterase 1
SNCA	alpha-synuclein, α-Syn
SPM	specialized pro-resolving mediator
SYNJ1	synaptojanin 1
TG	triglyceride
ω-3 PUFAs	omega-3 polyunsaturated fatty acids
ω-6 PUFAs	omega-6 polyunsaturated fatty acids
